# Genetics, shared environment, or individual experience? A cross-sectional study of the health status following SARS-CoV-2 infection in monozygotic and dizygotic twins

**DOI:** 10.3389/fpsyt.2022.1048676

**Published:** 2022-11-24

**Authors:** Sophia Kristina Rupp, Katja Weimer, Miriam Goebel-Stengel, Paul Enck, Stephan Zipfel, Andreas Stengel

**Affiliations:** ^1^Department of Psychosomatic Medicine and Psychotherapy, University Hospital Tübingen, Tübingen, Germany; ^2^Department of Psychosomatic Medicine and Psychotherapy, Ulm University Medical Center, Ulm, Germany; ^3^Clinic for Internal Medicine, Helios Clinic Rottweil, Rottweil, Germany; ^4^Department for Psychosomatic Medicine, Charité Center for Internal Medicine and Dermatology, Humboldt-Universität zu Berlin and Berlin Institute of Health, Charité-Universitätsmedizin Berlin, Berlin, Germany

**Keywords:** functional, general symptoms, mental health, psychosomatic, somatoform

## Abstract

**Background:**

The clinical presentation of COVID-19 shows a remarkably broad spectrum of symptoms. Although studies with adult twins on SARS-CoV-2 infection are rare so far, the fact that there is a genetic component associated with the highly variable clinical outcomes of COVID-19 has already been highlighted in recent studies investigating potential candidate genes and polymorphisms. This is the first study of adult monozygotic (MZ) and dizygotic (DZ) twins concordantly affected by SARS-CoV-2 infection to estimate variances explained by genetic, shared, and individual environmental components of both somatic and psychological symptoms following SARS-CoV-2 infection.

**Materials and methods:**

Data were collected from 10 adult twin pairs (5 MZ, 5 DZ) in which both twins already had a SARS-CoV-2 infection. A self-designed questionnaire, the Barthel Index, and the Multidimensional Fatigue Inventory (MFI) were used to assess various symptoms and health status following SARS-CoV-2 infection. Intra-class correlations were calculated, and the Falconer formula was used to quantify and differentiate the percentages of genetic influences as well as common environment and personal experiences on the examined traits. In addition, potential factors influencing symptom burden were examined and discussed.

**Results:**

We found high estimated heritability for mental impairment after SARS-CoV-2 infection (*h*^2^ = 1.158) and for general fatigue (*h*^2^ = 1.258). For symptom burden, reduced activity, and reduced motivation the individual environment appears to have the strongest influence. Other fatigue symptoms are influenced by genetic effects which range between 42.8 and 69.4%.

**Conclusion:**

Both genetics and individual environment play a role in health status after SARS-CoV-2 infection–mental status could be influenced primarily by genetic make-up, whereas for symptom burden and certain fatigue dimensions, non-shared environment could play a more critical role. Possible individual factors influencing the course of the disease were identified. However, gene-environment interactions may still be a source of differences between twins, and the search for candidate genes remains crucial on the road to personalized medicine.

## Introduction

The ongoing pandemic of coronavirus disease 2019 (COVID-19) poses a major public health challenge worldwide. COVID-19 is the disease caused by the SARS-CoV-2 virus, which was first detected in China in December 2019 (1). The ensuing pandemic had an immeasurable impact on humanity, claiming over 6 million lives until August 2022. The clinical presentation of COVID-19 shows a remarkably wide spectrum, from asymptomatic to acute respiratory distress syndrome, chronically persistent symptoms and death (2). As potential therapeutics and vaccines are developed and tested, it becomes increasingly important to identify potential candidate genes or polymorphisms along with influencing factors that could affect disease progression and health outcomes.

Twin studies are an important methodological tool for recording genetic and environmental influences. Monozygotic twins (MZ) have an almost identical genetic constitution and are also exposed to a comparable influence of environmental factors when they grow up together. Dizygotic twins (DZ), who grow up together, are equally exposed to a comparable influence of environmental factors, but in contrast to MZ share only about 50% of their genes (3). Therefore, comparing traits between MZ and DZ may help to capture the determining part of genetics and environmental factors. The study of concordance and discordance in MZ compared to DZ is one of the methodological ways to determine the contribution of genes to disease genesis and progression, in addition to an elaborate study of the human genome of the twin pairs. When a disease is mainly dependent on the environmental setting, MZ and DZ can be expected to be equally affected. On the other hand, when the host genome plays an important role, the concordance of results is higher in MZ than in DZ (3).

It is already known that infectious diseases in general may have a heritable component. More precisely, the susceptibility to contract an active infection as well as the severity of the immune response may depend on host genetic components (4). In the example of influenza due to H1N1 infection, immunogenetic factors have been shown to play a role regarding the risk and severity of infection (5). Other viral infections where genes are known to contribute to infection susceptibility or severity include hepatitis-B (6) and HIV–where heritability to infection susceptibility is estimated to be 28–42% (7). Other evidence that hosts genetic make-up influences susceptibility to infectious diseases comes from twin studies: Here it has been reported that host genetic factors may play an important role in susceptibility to infectious diseases such as poliomyelitis, tuberculosis, leprosy, infectious mononucleosis, and hepatitis B (8–13). Genetic variations in immune response or antigen recognition might be a biologically plausible mechanism for heritability. Taking genetic susceptibility to primary EBV infection as an example, several studies have found that heritable factors lead to differences in cytokine production, antigen recognition, and immune response (14–16).

However, only less is known about the host genetic factors that influence human infection with coronaviruses in general. Overall, studies with adult twins on SARS-CoV-2 infection are scarce but have already highlighted the fact that there is a genetic component associated with the highly variable clinical outcomes of SARS-CoV-2 infection. Since the corona pandemic, there have been a few case reports of twins contracting the virus and the course of the disease (17–19). In a large twin study from the UK, a special app was used to investigate the heritability of clinical manifestations of acute SARS-CoV-2 infection. The actual infection was predicted by the app, so the researchers point out that the results may suffer from healthy volunteer bias. Nevertheless, the results provide clues for planning genome-wide association studies to identify specific genes involved in viral infectivity and the host immune response (20). In addition, a recent paper examined concordance rates in 10 pairs of young twins (0–30 years old) and found a higher concordance rate in the MZ group, further supporting the potential role of genetic influences in the variable clinical manifestations of COVID-19 (21).

Therefore, there is an urgent need for more twin studies to further investigate the complex multifactorial inheritance and environmental influences that affect susceptibility and resistance to SARS-CoV-2 and the course of infection. Thus, we conducted this cross-sectional study to assess the variances in somatic and psychological symptoms following SARS-CoV-2 infection explained by genetic, shared environmental and individual components, investigating adult MZ and DZ with concordant SARS-CoV-2 infection.

## Materials and methods

### Participants and procedure

The study sample is derived from the German TwinHealth Twin Registry at the University Hospital of Tübingen (22), which currently contains information on more than 400 adult twin pairs of different ages and geographical areas who gave their written consent to be contacted to participate in TwinHealth research projects.

Inclusion criteria for this study were fluency in German and participation of both twins in the online survey. The current sample includes 155 twin pairs who participated in a COVID-19 online survey in early 2022. The survey included questions about symptoms since SARS-CoV-2 infection and effects of the infection on the physical and mental health of adult twins. Of these 155 twin pairs, 10 pairs reported that both twins were already positive for SARS-CoV-2.

This study was approved by the ethics committee of the University of Tübingen (project No. 174/2020BO1) and was conducted in accordance with the Declaration of Helsinki.

### Zygosity assessment

Zygosity was assessed using questions on similarity of appearance between twins (e.g., hair color, eye color, and overall appearance), confusion by strangers, and previous genetic zygosity tests. It has been shown that MZ and DZ can be reliably distinguished by this method (23, 24). A zygosity score between 0 (high dissimilarity) and 20 (high similarity) was calculated (25). A score of ≥10 or indicated MZ, while a score of <10 was indicative of DZ. The scores were compared with the self-report on the zygosity of the twins and agreed in all cases.

### Measures

A self-designed questionnaire (see Supplementary material) was used which contained questions on socio-demographic characteristics, current height and weight, and smoking behavior. Furthermore, the date of survey participation, the date of the infection and the current vaccination status were assessed. In addition, current complaints and physical limitations that have existed since the SARS-CoV-2 infection or have been attributed to the infection were collected. For each selected symptom, one point was assigned and a new variable for symptom burden was calculated by adding all points. Moreover, the mental impairment due to the infection was rated from “0 = not at all” to “10 = very strongly.”

The Barthel Index was used to assess independence in relation to basic everyday functions since the infection. This test asks for 10 different activity areas of daily living, each with 2–4 scoring options, which are then assessed with points (26). The range is 0–100 with 100 points signifying no impairment in everyday life with complete autonomy.

The Multidimensional Fatigue Inventory (MFI) was used to assess the severity and various domains of the fatigue syndrome. The instrument consists of 20 items on 5 dimensions of the fatigue syndrome: General fatigue, physical fatigue, mental fatigue, reduced activity, and reduced motivation (27). For each of the 20 items, the respondent is offered 5 response options ranging from “Yes, that is true” to “No, that is not true.”

### Statistical analyses

For the descriptive analyses of the collected data and the description of the sample, statistical measures such as mean with the associated standard deviation, minimum, and maximum were reported for metric variables. Absolute and relative frequencies were determined for categorical variables. Normal distribution of variables was assessed using the Kolmogorov–Smirnov test and visual inspection of the data with quantile-quantile plots. Differences of categorical variables with an expected cell frequency of <5 were determined using Fisher’s Exact Test.

Twin data were arranged according to the registration order at the TwinHealth Registry, i.e., the twin registered first was assigned the suffix A, while the other twin was assigned B accordingly.

For more precise quantification and differentiation of percentage shares of genetic influences, as well as common environment and personal experiences on the examined traits, intra-class correlations (ICC, one-way random, and single measurement) were calculated separately for MZ and DZ (28, 29). ICCs are a measure for estimating inter-rater reliability in the assessment of an outcome or characteristic (28). ICCs generally range from 0 to 1, but like any correlation, can take values between −1 and 1 (−1 < ICC < +1). ICCs were interpreted as follows: <0.40 = poor, between 0.40 and 0.74 = moderate to good, between 0.75 and 1.00 = excellent (30). ICCs take on negative values when the variance within twin pairs is higher than the variance between pairs. Negative ICCs should be accordingly interpreted as no correlation (31) and are assumed to be zero in subsequent calculations using Falconer’s formula (28, 29). The Falconer’s formula is used to determine the percentages of genetic and environmental influence (32). The theoretical assumptions of this model are as follows: (1) MZ share 100% of their genes; (2) DZ share 50% of their genetic material; (3) MZ and DZ growing up together share 100% of their common environment; (4) Other effects such as non-shared environment, individual learning experiences, and measurement errors contribute to differences within twin pairs. Based on the calculated twin correlation, heritability [*h*^2^ = 2 *(rMZ–rDZ)], shared environmental effects (*c*^2^ = 2 *rDZ–rMZ) and non-shared or individual environmental effects (*e*^2^ = 1–rMZ) are estimated using the Falconer’s formula. Consequently, the relative influences of heritability and shared and individual environment add up to 100%. Consequently, high correlations within MZ, which are at the same time higher than correlations within DZ, indicate the presence of a genetic effect. When correlations are high in both MZ and DZ, shared environmental influences play a major role, whereas when correlations are low, non-shared or individual environmental influences are responsible for the twins’ dissimilarity.

All statistical analyses were performed with IBM SPSS Statistics for Windows, version 27.0 (IBM Corp., Armonk, NY, USA). Significance level was set at *p* < 0.05 for all analyses.

## Results

### Study population

The study population included 310 twins (81 MZ and 74 DZ pairs). Of 81 MZ pairs, 65 pairs were female, and 16 pairs were male. The 74 DZ pairs included 39 female, 8 male, and 27 opposite-sex pairs. MZ were 43.8 ± 15.8 years old, DZ were 43.8 ± 16.6 years old (Table 1).

**TABLE 1:** Demographics and baseline characteristics of the study population.

Gender		
	***n***	**%**

Female	235	75.81
Male	75	24.19

**Zygosity**		

	***n***	**%**

Monozygotic	162	52.26
Dizygotic	148	47.74

**Age (years)**		

	**Monozygotic**	**Dizygotic**

Mean	43.8	43.8
SD	15.8	16.6
Minimum	18	19
Maximum	77	82

The actual study sample comprised 20 twins (5 MZ and 5 DZ pairs) concordantly infected with SARS-CoV-2. Of 5 MZ pairs, all pairs were female, while 5 DZ pairs included 4 female and 1 opposite-sex pair. MZ were 36.0 ± 12.6 years old, DZ were 43.6 ± 16.9 years old. Of the 10 pairs, one pair lived in the same household.

### Common symptoms within pairs

A Fisher’s Exact Test was performed between current complaints and limitations of twin A and current complaints and limitations of twin B. There were no statistically significant correlations between current complaints and limitations within twin pairs (Table 2).

**TABLE 2:** Frequency of common symptoms following SARS-CoV-2 infection within pairs.

Variables	Twin A (*n*)	Twin B (*n*)	*P*-value (2-sided)
**Pulmonary symptoms**			
Shortness of breath/dyspnea	3	1	>0.999
Cough	4	4	0.571
Scratchy throat/sore throat	3	3	>0.999
**Cardiac symptoms**			
Heart stuttering, palpitations, cardiac arrhythmia, blood pressure fluctuations	1	1	>0.999
**Gastrointestinal symptoms**			
Gastrointestinal complaints	1	2	0.200
**Neurological symptoms**			
Pain in limbs, muscles	0	4	–
Smelling, olfactory disorder	0	0	–
Taste disorder	0	0	–
Lack of strength/general weakness	2	3	>0.999
Headache	1	5	>0.999
**Psychological symptoms**			
Fatigue, tiredness, sleepiness	4	6	0.571
Sleeping disorders	3	2	>0.999
Anxiety disorders	0	0	–
Depressive mood	1	2	>0.999
Mood swings	1	0	–
Aggressiveness	0	0	–
Increased irritability	1	0	–
**Other symptoms**			
Hair loss	0	0	–
No complaints	1	3	>0.999
Others	3	1	>0.999
**Restrictions in daily activities**			
During personal hygiene, household etc.–dyspnea	0	0	–
During personal hygiene, household etc.–shortness of breath	2	0	–
During personal hygiene, household etc.–lack of strength	2	3	>0.999
When walking on level ground–dyspnea	0	0	–
When walking on level ground–shortness of breath	0	0	–
When walking on level ground–lack of strength	1	3	0.300
When walking one floor–dyspnea	0	1	–
When walking one floor–shortness of breath	2	1	>0.999
When walking one floor–lack of strength	2	3	>0.999
During sport–dyspnea	1	1	>0.999
During sport–shortness of breath	3	1	0.300
During sport–lack of strength	1	2	>0.999
10 items of the Barthel Index	0	0	–

*n* = 20, Fisher’s Exact Test.

Symptom burden after SARS-CoV-2 infection significantly correlated within DZ, but not between MZ. There were no statistically significant correlations of fatigue symptoms or mental impairment between MZ or DZ. Regarding the Barthel index, all twins scored the same (variance = 0), therefore the correlation should be interpreted as 1 (Table 3).

**TABLE 3:** Impairments related to SARS-CoV-2 infection in MZ and DZ twin pairs (reported as mean ± standard deviation) and intra-class correlations (reported as ICC coefficients and 95% CI).

Item	MZ pairs (*n* = 5)			DZ pairs (*n* = 5)		
	Twin A	Twin B	ICC [95% CI]	Twin A	Twin B	ICC [95% CI]
Mental impairment after SARS-CoV-2 infection	2.4 ± 1.3	1.6 ± 1.3	0.579 [−0.327 to 0.945]	1.2 ± 0.4	2.6 ± 2.6	−0.139 [−0.814 to 0.752]
Symptom burden after SARS-CoV-2 infection	1.8 ± 1.3	3.0 ± 2.6	−0.516 [−0.917 to 0.498]	3.8 ± 3.8	3.8 ± 3.6	0.749* [−0.030 to 0.970]
General fatigue (MFI)	10.2 ± 3.1	11.2 ± 3.4	0.629 [−0.254 to 0.953]	9.8 ± 6.1	11.6 ± 2.2	−0.398 [−0.890 to 0.602]
Physical fatigue (MFI)	7.8 ± 2.8	10.2 ± 3.6	0.347 [−0.564 to 0.901]	11.2 ± 5.9	10.0 ± 5.1	−0.361 [−0.880 to 0.630]
Reduced activity (MFI)	10.2 ± 4.0	8.6 ± 3.5	−0.055 [−0.784 to 0.787]	8.0 ± 2.4	9.2 ± 5.2	0.055 [−0.737 to 0.825]
Reduced motivation (MFI)	7.6 ± 1.1	7.8 ± 4.8	0.142 [−0.695 to 0.852]	8.0 ± 4.8	8.4 ± 3.4	0.165 [−0.683 to 0.858]
Mental fatigue (MFI)	10.0 ± 5.8	8.6 ± 4.3	0.629 [−0.255 to 0.952]	9.8 ± 2.2	11.0 ± 4.3	0.315 [−0.587 to 0.895]
Total fatigue score (MFI)	45.8 ± 10.5	46.4 ± 15.0	0.214 [−0.654 to 0.871]	46.8 ± 18.2	50.2 ± 19.3	−0.286 [−0.860 to 0.678]
Barthel Index	100 ± 0.0	100 ± 0.0	–	100 ± 0.0	100 ± 0.0	–

*p < 0.05. DZ, Dizygotic twins; ICC, Intra-class correlation coefficient; MFI, Multidimensional fatigue inventory; MZ, Monozygotic twins. Significant correlations are shown in bold.

### Genetic, common, and individual environment contributions to outcomes following SARS-CoV-2 infection

**FIGURE 1:**
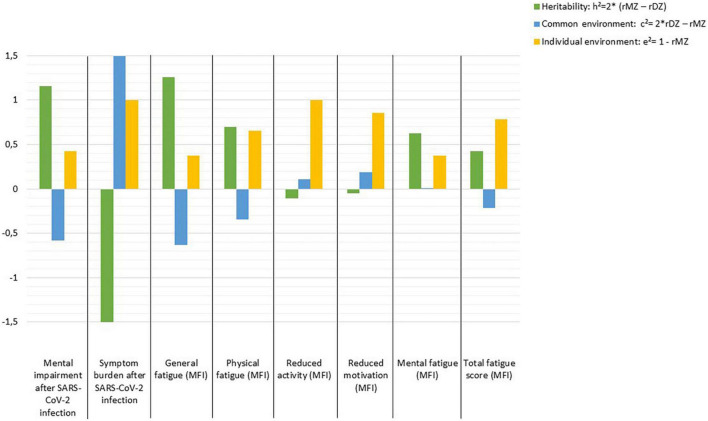
Estimates of heritability (h^2^), common (c^2^), and individual environmental (e^2^) effects on outcomes following SARS-CoV-2 infection according to Falconer’s formula.

High heritability (*h*^2^ = 1.158) is estimated for mental impairment after SARS-CoV-2 infection and *h*^2^ = 1.258 for general fatigue, whereas for symptom burden, reduced activity, and reduced motivation the individual environment appears to have the strongest influence, while there seems to be no evidence of heritability. Other fatigue symptoms are influenced by genetic effects which range between *h*^2^ = 0.428 and *h*^2^ = 0.694. In contrast, non-shared or individual environmental effect on total fatigue score is estimated as *e*^2^ = 0.786 (Figure 1).

### Influencing factors on symptom burden

Of 20 twins, there were 3 (15%) current smokers, while 17 (85%) were non-smokers. All 3 smokers belonged to 3 different twin pairs. There was no pair of concordant current smokers. A Fisher’s Exact Test was performed for smoking status of twin A and smoking status of twin B and showed no statistically significant relationship within pairs with a *p*-value (two-sided) of >0.999. The smoking status displayed a negative correlation with symptom burden without statistical significance. Since smoking status is coded as “smoker” with a 1 and “non-smoker” with a 2, this point biserial correlation thus indicates that subjects who currently smoke tend to show a higher symptom burden than non-smokers (Table 4).

**TABLE 4:** Correlations between smoking behavior, body-mass-index, vaccination status, time interval (between survey participation and infection), and symptom burden.

Item	Symptom burden
Smoking behavior^a^	–0.334
Body mass index^b^	0.128
Vaccination status^b^	0.563*
Time interval^b^	–0.333

*p < 0.05; ^a^calculated as Pearson’s correlation; ^b^calculated as Spearman’s correlation. Significant correlations are shown in bold.

The mean BMI of the 10 pairs was 23.6 ± 5.3 kg/m^2^, while the minimum was 19.2 kg/m^2^ and the maximum 38.1 kg/m^2^. The BMI of twin A and twin B and showed a statistically significant positive correlation with each other (Spearman’s ρ = 0.729; *p* = 0.017). The BMI and current symptom burden correlated slightly positively, but not significantly with each other (Table 4).

Regarding vaccination status, of 20 twins, 3 (15%) had not been vaccinated against SARS-CoV-2, 2 (10%) had been vaccinated one time, 2 (10%) had already received two vaccinations, and 13 (65%) twins had been vaccinated three times. Vaccination status of twin A and vaccination status of twin B exhibited a strongly significant positive correlation with each other (Spearman’s ρ = 0.885; *p* = 0.001). The vaccination status and the currently existing symptom burden correlated statistically significantly positively with each other (Table 4).

The time interval between survey participation and infection averaged 62.9 ± 125.2 days. The minimum time interval was 1 day, the maximum 702 days. Between the time interval (survey and infection) of twin A and twin B, there was no significant correlation with each other (Spearman’s ρ = −0.061; *p* = 0.868). The time interval and current symptom burden did not correlate significantly with each other (Table 4).

## Discussion

To the best of our knowledge, this is the first study with adult MZ and DZ concordantly affected with SARS-CoV-2 infection estimating the variances explained by genetic, common, and individual environmental components of both somatic and mental symptoms following SARS-CoV-2 infection.

Symptoms following SARS-CoV-2 infection, psychological impairment since infection and fatigue were assessed by having both twins participate in the survey at one time point. Our results predominantly show no statistically significant correlations between current complaints and limitations within twin pairs after SARS-CoV-2 infection. These findings are in line with recent case reports of MZ who showed very different disease courses in the context of COVID-19 (17–19).

Our results further show moderate to good correlations within MZ pairs for mental impairment after SARS-CoV-2 infection, general and mental fatigue. In addition, our results indicate good correlations within DZ pairs for symptom burden related to COVID-19. The moderately high intra-pair correlations in MZ suggest that the influence of genetics as well as shared environmental components is high, and that the individual environment may play a lesser role. The estimates of heritability (h^2^), common (c^2^) and individual (e^2^) environmental influences confirm our findings: High heritability was found for mental impairment, as well as general and mental fatigue. However, the imperfection of this model for estimating variances explained by genes, shared and individual environment is evident, as these estimates for mental impairment add up to more than 100%, as the shared environment (c^2^) was estimated negatively and should be subtracted. Accordingly, the calculations of h^2^, c^2^, and e^2^ should be interpreted as estimates indicating the direction of the effects, but not as absolute values. In line with our findings, another study on adult twins also describes a heritability of 31% for fatigue symptoms (20). However, this study recorded symptoms of acute infection and the actual infection was predicted by the app, so the researchers suggest that the results may suffer from bias from healthy volunteers (20).

Our results for symptom burden, reduced activity, reduced motivation, and total fatigue point to a high influence of non-shared or individual environment. Interestingly, a 2022 study reported candidate genes that may influence the severity of COVID-19 (33). There are also results suggesting the importance of host genetics for the risk of clinical manifestations of COVID-19 (20, 34) and for a more likely severe course of the disease (35). Results from a Brazilian study of young twin pairs (0–30 years old) showed a higher concordance rate in MZ, while the discordance rate was higher in DZ. These results also suggest a multifactorial inheritance that could modulate susceptibility or resistance to SARS-CoV-2 infection. Since the study has not been peer-reviewed yet, the results should be interpreted with caution (21). Overall, it can be stated that planning and conducting genome-wide association studies to identify specific genes involved in viral infectivity and host immune response will help to further shed light on this issue.

As discussed above, in our study the reported symptoms frequently differed within twin pairs and the overall symptom burden was rather low. We therefore took a closer look at possible individual factors influencing the course of the disease: Firstly, it is noticeable that 95% of our study population was female. In terms of gender, there are studies indicating female gender as a risk factor for longer-lasting symptoms following SARS-CoV-2 infection (36, 37). On the other hand, there seems to be no difference in susceptibility to infection, but many studies report male gender as a risk factor for more severe COVID-19 (38–42), which could explain the rather low symptom burden in our study population.

Other risk factors for prolonged symptoms after SARS-CoV-2 infection include older age, white ethnicity, and obesity (36). In our study, the mean BMI of the twin pairs was 23.6 kg/m^2^. We also found that BMI was interdependent within pairs. Further, our findings indicate that higher BMI may be associated with higher symptom burden, which is consistent with several other studies pointing to obesity as a risk factor for more severe disease progression (39, 43–47).

We further assessed the current smoking status of the participants. A total of 85% were non-smokers, while 15% were current smokers. Smoking status within pairs was independent of each other, which could explain why symptoms differed within pairs. Our analyses indicated that subjects who were current smokers tended to show a higher symptom burden than non-smokers. In line with our findings, current evidence suggests that smoking may worsen COVID-19 outcomes (39, 48, 49). However, a recent case report described a possible mechanism for nicotine to attenuate the subsequent inflammatory process of COVID-19 (19); this potential mechanism needs further investigation.

Vaccination status could also inform us about the different symptoms within twin pairs and the course of COVID-19. Vaccinated individuals were shown to have a lower risk of developing symptomatic SARS-CoV-2 infection (50) or developing COVID-19 sequelae in the one to 6 months period following infection compared with unvaccinated individuals (51) which is inconsistent with our findings. Of 20 twin pairs participating in our study, 15% had not been vaccinated against SARS-CoV-2 and 85% had already received one or more vaccinations. Vaccination status was found to be interdependent within pairs. Surprisingly, the number of SARS-CoV-2 vaccinations and the current symptom burden were significantly positively correlated. In our study, we assessed the current vaccination status of the twins, but not the vaccination status at the time of infection or the exact date of vaccination, so it is unclear how much time there was between vaccination and infection and whether participants have been vaccinated after infection. This additional information could help in the interpretation of our results, as the probability of longer-lasting symptoms seems to depend on the time since vaccination (52).

Furthermore, we took a closer look at the period between SARS-CoV-2 infection and survey participation. Here we found that the average time interval was 62.9 days. Moreover, the time intervals differed within the twin pairs, and twins with a greater time interval between infection and participation in the survey tended to have lower symptom burden scores. These observations are consistent with recent studies and may also explain the rather low current symptom burden in our study population and the symptom variation within pairs. More precisely, recent analyses showed that symptoms in patients with COVID-19 lasted an average of 2.5 months and persisted for an average of 1.5 months after recovery (53). Other findings suggest an absence of symptoms in non-hospitalized patients within the first 1.5–6 months after the onset of symptoms (54). The authors reported that an improvement in symptoms occurs mainly in the first weeks after the acute phase (54). The most common current persisting symptoms reported by the twins in our study were cough and sore throat, as well as fatigue and headache. Similarly, other studies reported fatigue (55–57) and headache (55) as the most common persisting symptoms.

Finally, some limitations of this study should be noted: The exclusive use of self-reports in absence of direct contact and physical examination of the patients, the small sample size, and the availability of data from only one point in time. Further, we did not determine zygosity by genetic testing, but relied on the twins’ own information about genetic testing, questions about the similarity and dissimilarity of the twins, and compared the results with the twins’ self-reported zygosity. However, this procedure showed high agreement with genetic tests (23, 24), but is of course not as precise as genetic tests. In addition, we did not assess comorbidities or symptoms at the time of the acute infection, although comorbidities and a high symptom burden during the acute phase are associated with persistent symptoms (54). Moreover, we did not record which COVID-19 vaccine was used, nor did we ask about or identify the SARS-CoV-2 variants, although it is known that the overall clinical manifestations differ between virus variants (58). For example, the Omicron variant is less likely to cause long-lasting symptoms than the Delta variant (52). Since in our study the discovery date was early 2022 in 80% of the cases (16 twins), we suspect an Omicron virus variant in at least these cases. Lastly, our sample consisted almost entirely of female participants, while no male twin pairs were represented in our analyses.

The strengths of our study are the following: First, our study cohort included only twin pairs concordant for SARS-CoV-2 infection allowing an assessment of genes and environmental influences. Second, all twins had a positive COVID-19 test, e.g., by PCR, so there is no healthy volunteer bias to be concerned about.

In summary, we have shown that both genetics and individual environment play a role in health conformation following COVID-19. We also identified possible individual factors influencing the course of the disease, namely BMI, smoking status, vaccination status, and time since infection. However, gene-environment interactions may still be a reason for differences between twins, and the search for candidate genes remains crucial on the road to personalized medicine. Future studies with larger sample sizes and longitudinal designs are needed, which should combine twin studies and genetic analyses.

## Data availability statement

The raw data supporting the conclusions of this article will be made available by the authors, without undue reservation.

## Ethics statement

The studies involving human participants were reviewed and approved by the Ethics Committee of the University of Tübingen. The patients/participants provided their written informed consent to participate in this study.

## Author contributions

AS, MG-S, and PE planned the study and gave critical input throughout the study. SR performed the study, organized the database, and wrote the first draft of the manuscript. SR and KW performed the statistical analyses. All authors contributed to the interpretation of data and all revisions and reviewed and finalized the manuscript.
