# Supplementary material for: Genetics, shared environment, or individual experience? A cross-sectional study of the health status following SARS-CoV-2 infection in monozygotic and dizygotic twins
Source: Front Psychiatry. 2022 Nov 24;13:1048676. doi: 10.3389/fpsyt.2022.1048676 (PMC9729738; doi:10.3389/fpsyt.2022.1048676)
Supplement: Supplementary file 1 [file Data_Sheet_1.pdf]

COVID-19 QUESTIONNAIRE – Twin
(please tick as appropriate)

Age:____, Gender (f/m/d):_____, Height (cm):____, Weight:_______(kg)

1. Smoking behavior:

How many packs per day do you/did you smoke:____ (packs/day) over how many years:_____ (years), ☐ until today / ☐ until ___ years ago

2. Have you had a corona disease?

yes ☐ no ☐

Detection by means of:

PCR ☐ Rapid test ☐ Antibodies ☐

Detection date: _________________ unknown ☐

3. If question 2 is filled in with yes: Do you CURRENTLY suffer from complaints that you attribute to your corona disease? Multiple choices are possible.

**[1]** ☐ Shortness of breath/ dyspnoea	**[2]** ☐ Pain in limbs, muscles	**[3]** ☐ Cough	**[4]** ☐ Scratchy throat/ sore throat
**[5]** ☐ Smelling, olfactory disorder	**[6]** ☐ Taste disorder	**[7]** ☐ Gastrointestinal complaints	**[8]** ☐ Lack of strength/ general weakness
**[9]** ☐ Tiredness, fatigue, sleepiness	**[10]**☐ Headache	**[11]** ☐ Heart stuttering, palpitations, cardiac arrhythmia, blood pressure fluctuations	**[12]** ☐ Hair loss
**[13]** ☐ Sleeping disorders	**[14]** ☐ Anxiety disorders	**[15]** ☐ Depressive mood	**[16]** ☐ mood swings
**[17]** ☐ Aggressiveness	**[18]** ☐ Increased irritability	**[19]** ☐ others: ________	☐ no complaints

4. If question 2 is filled in with yes: Do you feel any restrictions in the following activities (shortness of breath/dyspnoea, reduced performance, etc.)?

During personal hygiene, household etc.	When walking on level ground:	When walking one floor:	During sport:
☐ Dyspnoea	☐ Dyspnoea	☐ Dyspnoea	☐ Dyspnoea
☐ Shortness of breath	☐ Shortness of breath	☐ Shortness of breath	☐ Shortness of breath
☐ Lack of strength	☐ Lack of strength	☐ Lack of strength	☐ Lack of strength

5. If question 2 is filled in with yes: How much do you feel psychologically affected by the corona disease?

Not at all very strong

☐ 0 ☐ 1 ☐ 2 ☐ 3 ☐ 4 ☐ 5 ☐ 6 ☐ 7 ☐ 8 ☐ 9 ☐ 10

6. Have you been vaccinated against SARS-CoV-2?

No ☐ yes, 1x ☐ yes, 2x ☐ yes, 3x ☐
